# First trimester placental endothelial cells from pregnancies with abnormal uterine artery Doppler are more sensitive to apoptotic stimuli

**DOI:** 10.1038/s41374-018-0139-z

**Published:** 2018-10-05

**Authors:** Nicoletta Charolidi, Amanda J. Host, Sandra Ashton, Zoe Tryfonos, Karin Leslie, Baskaran Thilaganathan, Judith E. Cartwright, Guy S. Whitley

**Affiliations:** 1 Centre for Vascular Biology, Institute of Molecular and Clinical Sciences, St George’s, University of London, Cranmer Terrace, London, SW17 0RE UK; 2 Department of Biology, Sevenoaks School, Sevenoaks, Kent, TN13 1HU, UK; 3 Fetal Medicine Unit St George’s Hospital Foundation Trust Blackshaw Road London, London, UK

**Keywords:** Experimental models of disease, Apoptosis

## Abstract

Failure of the placental capillary network to develop normally is associated with early onset fetal growth restriction (FGR) and pre-eclampsia (PE). Although the symptoms are observed at term, the problem begins in the first trimester. However, investigations at this clinically relevant time are hindered by difficulties in identifying earlystage pregnancies that are at risk of developing FGR/PE. Using uterine artery Doppler ultrasound in the first trimester as a proxy measure of poor placentation, we have identified pregnancies at increased risk of developing early onset FGR/PE. Placental endothelial cells (PEC) isolated from pregnancies at increased risk of developing FGR/PE grew more slowly and their basal rate of apoptosis was significantly higher than that seen in the normal group. The pro-apoptotic stimulus, TNFα, induced apoptosis in cells from both groups but this was significantly greater in the high risk group. TNF receptor expression was unaffected. Inhibition of nitric oxide (NO) production significantly increased the sensitivity of cells from the normal pregnancies to TNFα but not in the high risk group establishing a functional role for NO in this system. In conclusion, first trimester PEC from pregnancies at increased risk of developing early onset FGR/PE were inherently more sensitive to apoptotic stimuli and this was functionally linked to the synthesis of NO. This may contribute to the poor placental vascular development seen in on going pregnancies.

## Abstract (web summary)

A poorly developed placental capillary network is associated with early onset fetal growth restriction (FGR) and pre-eclampsia (PE). First trimester placental endothelial cells from pregnancies at increased risk of developing early onset FGR/PE were more sensitive to apoptotic stimuli and this was functionally linked to the synthesis of NO. This may contribute to the poor placental vascular development seen in ongoing pregnancies.

## Introduction

Fetal growth restriction (FGR) is a common pregnancy complication whereby the baby fails to reach its genetically determined growth potential. It affects 5-8% of births worldwide and is a major cause of neonatal morbidity and mortality. FGR is often but not always associated with pre-eclampsia (PE). Fetal weight correlates with placental weight and the placental weight of babies born with FGR is 24% less than that of an infant born with an appropriate weight for their age [1, 2]. Although it was once thought that poor placental development was purely a pregnancy related disorder, there is now increasing evidence to support the idea that failure to thrive in utero, as a result of placental insufficiency, increases the risk of developing cardiovascular and metabolic diseases both in childhood and in later life [3].

The placenta is the interface between the mother and the developing foetus, transferring oxygen and nutrients from maternal blood. To facilitate this, a functioning placental circulation with a highly efficient network of capillaries within the placental villi is required. Vasculogenesis, and subsequent angiogenesis, are responsible for the enlargement of the placental vascular tree and placental growth. Vessels develop from 18-20 days post-conception when precursor endothelial cells (EC), derived from the mesoderm, form cords of cells beneath the trophoblastic epithelium. By 6 weeks post-conception, a villous circulation has formed. In the first and early second trimesters, the number, volume and surface area of the placental capillaries expands with dramatic increases in capillary length occurring through proliferation and remodelling until term. Both branching and non-branching angiogenesis occur during placentation; however branching angiogenesis predominates in early pregnancy [4]. Vascular casts from pregnancies affected by early onset FGR and PE have demonstrated striking differences in placental vessels compared with normal pregnancies [4].

The aetiology of early onset FGR with or without PE is believed to occur in the first trimester, yet to date, developmental and functional studies on human placental endothelial cells (PEC) have used cells isolated from term placentae [5–7]. Although there are significant advantages to this approach such as the abundance of material and known pregnancy outcome, it is not always possible to extrapolate these findings back to the first trimester. To try and overcome this we have used Uterine artery Doppler (UtAD) ultrasound. UtAD has been found to be predictive of placental complications in pregnancy. Detection of a high-resistance index (high-RI) by UtAD ultrasound is indicative of early placental insufficiency caused by reduced trophoblast invasion and poor spiral artery remodelling [8]. Using this screening method we have shown that pregnancies with a high-RI are five times more likely to develop early onset PE and/or FGR than those pregnancies with a normal-resistance index (normal-RI) [9].

In this study, we isolated PEC from first trimester terminations of pregnancies that have been screened by UtAD scanning and compared the sensitivity of cells isolated from pregnancies with a normal-RI and high-RI to the apoptotic stimulus TNFα. We further examined whether the difference in sensitivity observed could be attributed to differences in the TNF receptor expression and the production of nitric oxide (NO), a known EC survival factor.

## Materials and methods

### Doppler ultrasound scanning of the uterine arteries and tissue collection

Doppler ultrasound screening of uterine arteries was performed on women undergoing elective surgical termination of pregnancy at St George’s University Hospital, NHS Foundation Trust. Inclusion criteria included singleton pregnancy, gestational age 9–14 weeks, normal fetal anatomy and nuchal translucency thickness, and no known maternal medical condition or history of recurrent miscarriage. The gestational age was calculated by crown-rump length measurement. Doppler ultrasound was performed by a trained sonographer as described previously [10]. Following a study of 10,000 ongoing pregnancies, we have established reference ranges of the resistance indices. In this study, a high-RI is defined as a pregnancy with bilateral uterine artery notches and a mean RI ≥ 95th centile; while a normal-RI presents no uterine artery notches and a mean RI < 95^th^ centile. Wandsworth Local Research Ethics committee approval was in place and all women gave written informed consent (ref: 01.96.8 and 01.78.5). There was no significant difference in gestational age between the patient groupings.

### Isolation of first trimester placental endothelial cells

Products of conception were collected and the placental tissue was dissected from the decidua, connective tissue and/or blood clots. The cleaned placenta was then finely chopped, placed in a 50-ml tube and washed with HBSS. The tissue was centrifuged at 300×*g* for 2 min, and HBSS was removed slowly with a disposable Pasteur pipette. This step was repeated three times and the wet tissue was weighed. For breaking the placental tissue, a series of enzymatic digestions were used in combination with mechanical force applied through a Kwill (125 mm; Universal Hospital Supplies, Enfield, UK) attached to a 20-ml syringe. The number of times that the tissue was passed through the Kwill was defined by the softness of the sample. Early-gestation placentae were normally softer and therefore required less processing through the Kwill.

Based on the wet weight of the placenta, the first enzymatic solution was prepared: for every gram of tissue, 4 ml of HBSS containing 0.25% (v/v) Trypsin (Invitrogen, Thermo Fisher Scientific, MA,USA) 1.3 mg/ml Dispase (Invitrogen, Thermo Fisher Scientific) and 400 U/g DNase (Merck, MO, USA) were used. The enzymatic solution and sample were mixed by passing through the Kwill up to 4 times, and immediately incubated at 37 °C with constant agitation for 15 min. It was then drawn though the Kwill up to 4 times (again, the number of times through the Kwill was defined by the softness of the digesting placenta). This procedure was followed one more time and the tissue digest was filtered through a 100-μm-mesh filter (Falcon, Corning, NY, USA).

The undigested tissue, which at this point had a gel-like consistency, was collected from the mesh and transferred back to the 50-ml tube for a second series of enzymatic digestion. The second solution was also prepared based on the initial wet weight of the placenta. For every gram of tissue, 4 ml of HBSS containing 2 KU/g collagenase (Invitrogen, Thermo Fisher Scientific) and 400 U/g DNase were used. The enzymatic solution and undigested tissue were passed through the Kwill up to 4 times and incubated at 37 °C for 8 min. This step was repeated another 2 times. The resulting tissue digest was then filtered through a 70-μm-mesh filter (Falcon, Corning). The filtrate was then centrifuged at 300 *g* for 10 min resulting in a cell pellet. This was washed once with 10 ml HBSS, re-centrifuged under the same conditions (300×*g* for 10 min) and re-suspended in 2 ml of ice-cold PBS/0.1% BSA (Sigma-Aldrich) containing 17 µl of washed CD31 Dynabeads®CD31 beads (Invitrogen, Thermo Fisher Scientific) for every gram of initial wet tissue. At this stage cells and CD31 beads were incubated at 4 °C with rotational mixing for 20 min, as detailed by the manufacturer. Using a magnetic rack, the supernatant, which contained cells that did not express CD31 (i.e., stromal cells), was removed, and beads were then washed 5 times with ice-cold PBS/0.1% BSA. The magnetic isolated PEC were then plated in culture dishes coated with rat tail collagen type 1 (49.2 µg/ml; BD Biosciences, CA, USA), using PEC medium (McCoys 5 A supplemented with 50 µg/ml penicillin-streptomycin, 2 mM L-glutamine, 2.5 µg/ml amphotericin B, 25% (v/v) Male AB serum, 16 U/ml Heparin and 2 mg/ml MgSO_4_ (all from Sigma-Aldrich), 5 ng/ml VEGF (PeproTech, London, UK). A total of 215 placentae—110 of which were of normal-RI and 105 of high-RI—were digested for the isolation of PEC used for the work presented in this paper. PEC used for the experiments described were seeded into the experimental wells and used within 24 h of isolation without being passaged.

### Cell culture conditions

Following isolation cells were seeded directly into the experimental wells at a density of between 30–40% and cultured for 48 h in 5% CO_2_, 6% O_2_ and 89% N_2_. All experimental procedures were performed in 5% CO_2_, in air at 37 °C.

### Immunocytochemistry

PEC were routinely fixed at 24 and 72–96 h post isolation and stained for purity. Cells were washed once with PBS for 5 min and fixed with ice-cold methanol for 10 min. They were then washed once with PBS for 5 min and permeabilised using PBS/0.2% (v/v) Tween-20 (Sigma-Aldrich) at room temperature for 10 min. Another two washes with PBS for 5 min followed and cells were blocked with PBS/10% (v/v) goat serum (GS; Vector Labs, Peterborough, UK) for 20 min at room temperature. The fixed and blocked cells underwent 3–5-min long washes with PBS and incubated with the primary antibody or isotype-matched immunoglobulin control made up in PBS/1.5% (v/v) GS and 0.2% (v/v) Tween-20 for 1 h at room temperature. Cells were then washed three times in PBS for 5 min and incubated in the secondary antibody for 45 min at room temperature. Following, after three washes with PBS for 5 min, cells were incubated with streptavidin-fluorescein (3.3 µg/ml, Vector Labs) for 30 min in the dark, at room temperature. Cells were washed three times with PBS and 1–2 drops of DAPI-containing Vectashield® (Vector Labs) was added. Images were captured using an Olympus IX70 fluorescence microscope with an XC10 camera and Cellsens dimensions software (all from Olympus, Tokyo, Japan). PEC purity was determined by merging images of DAPI-stained nuclei and vWF-positive cells using Adobe Photoshop CS2 (Adobe Systems, CA, USA). The percentage of vWF-positive cells in the total cell population was calculated.

### Immunohistochemistry

Paraffin-embedded placental tissue sections (5 µm) were cut and mounted on slides and immersed twice in xylene for 5 min. The slides were then rinsed with 100% ethanol and re-hydrated with PBS using a series of ethanol dilutions (100% (v/v), 90, 80, 70, 50 and 30%). Antigen retrieval was achieved following incubation with proteinase K (20 µg/ml; Invitrogen, Thermo Fisher Scientific) for 30–40 mins. Slides were washed once with PBS and blocked with PBS/10% (v/v) GS for 20 min at room temperature. Immunolabelling of tissues was then performed using the same procedure as for immunocytochemistry. Slides were mounted with DAPI-containing Vectashield® prior to visualisation. Images were captured as above.

### PEC angiogenesis assay

The wells of Angiogenesis µ-Slides (Thistle Scientific, Glasgow, UK) were coated with 10 µl of collagen type 1 (3 mg/ml) as per manufacturer’s instructions. PEC were added to the wells at 6.3 × 10^5^ cells/ml in PEC medium (50 µl/well). The following day, the cells were washed with PBS and 50 µl fresh PEC medium was added. Cells were incubated at 37 °C (5% CO_2_ in air) for 6 days. Images were taken as above, and merged using Adobe Photoshop CS2.

### Proliferation and apoptosis assays

Twelve-well plates were coated with 49.2 µg/ml collagen type 1 (Corning) before seeding freshly isolated, non-passaged PEC with a density of 30–40%. After 48 h, the media were replaced with fresh PEC culture media containing the apoptotic stimuli 30 ng/ml TNFα (Sigma-Aldrich) and 800 ng/ml Actinomycin D (Sigma-Aldrich). In subsequent experiments, cells were incubated for 48 h fresh PEC culture media containing 30 ng/ml TNFα in the presence and absence of the nitric oxide synthase inhibitor, 500 µM L-NAME (Sigma- Aldrich). Cellular behaviour was monitored by time-lapse digital image microscopy using an Olympus IX70 inverted microscope equipped with a Hamamatsu C4742-95 digital camera as previously described for the times indicated [11–13].The microscope and stage were enclosed within a heated (37 °C) humidified atmosphere of 5% CO_2_ in air (Solent Scientific, Fareham, UK). Analysis was performed using Image Pro-Plus software (Media Cybernetics, MD, USA). At the beginning of each sequence, 40 cells from each treatment were randomly selected from a field of view. PEC were identified by the adherence of CD31 coated beads to the cell surface. Cells were tracked through the sequence and scored according to the frame at which the cells divided or showed an obvious apoptotic morphology (transition to a phase-bright appearance, decrease in cytoplasmic and nuclear size and formation of a membrane bleb or blister). From this data, kinetics curves were then produced for each patient. Using Graph-pad Prism software the area under the curve for each patient was determined and the mean of at least five individual patients analysed.

### cDNA and quantitative real-time PCR (qRT-PCR)

For the synthesis of cDNA, RNA was extracted from isolated PEC cultured for 96 h, using the RNeasy Plus Micro Kit (Qiagen), following the manufacturer’s recommendations. Genomic DNA was eliminated with the gDNA Eliminator spin column (Qiagen) and up to 2 µg of whole RNA was random-primed and reverse-transcribed to cDNA with the Tetro cDNA Synthesis Kit (Bioline, London, UK), according to the manufacturer’s instructions. For selection of the most stably expressed genes of placental cells at this stage of development, we selected phospholipase A2 (YWHAZ) and 18 S ribosomal-5-RNA (RNA 18S5) [14–17]. The relative quantities of NOS3, TNFRSF1A and TNFRSF1B gene transcripts were analysed by probe-based qRT-PCR (TaqmanTM gene expression assays, Thermo Fisher Scientific), prepared in duplicate from 6–7 normal- and 6–7 high-RI, gestionally-matched placental endothelial cell samples. Each qRT-PCR reaction was prepared with 25 ng (in 5 µl) of each cDNA sample, 1 µl of each of the TaqmanTM probes, 10 µl of TaqmanTM Gene Expression Master Mix (Thermo Fisher Scientific) and 5 µl of nuclease-free water. Products were amplified under the universal thermal conditions (50 °C for 2 min, 95 °C for 10 min, and then 40 cycles of 95 °C for 15 s and 60 °C for 1 min) using a CFX384 Touch cycler (Bio-Rad, CA, USA). The performance of the Taqman assay was verified to be 100% by generating a standard curve and calculating the PCR efficiencies (E = 10^(−1/slope)), for each of the TaqmanTM probes (NOS3, TNFRSF1A, TNFRSF1B, YWHAZ and RNA 18S5). Unknown samples were normalised against the average expression of the two reference genes and relative gene expression was calculated using the ΔΔCT method.

### Determination of cGMP in chorionic villous tissue

Chorionic villous tissue from 23 normal and 16 high risk pregnancies were snap-frozen in liquid N_2,_ finely ground with a mortar and pestle and then homogenised in 10 volumes of ice-cold 0.1 M HCl and centrifuged at 600×*g* for 10 min at 4 °C. cGMP was then detected by immunoassay following the manufactures instructions (R&D Systems, MN, USA). The results were expressed per mg of protein as determined by a Bradford assay.

### Determination of nitrite and nitrate in chorionic villous tissue

Chorionic villous tissue from 30 normal and 37 high risk pregnancies were snap-frozen in liquid N_2,_ and finely ground before homogenisation in ice-cold PBS. The samples were then centrifuged at 10,000×*g* at 4 °C for 20 min. The supernatant was removed and centrifuged at 100,000×*g* at 4 °C for further 30 min. The resulting supernatant was passed first through a 0.45 µm filter and then a 20 Kd molecular weight cut-off filter. Nitrate and nitrite was determined using a fluorometric assay kit following the manufacturer’s instructions (Cayman Chemicals, MI US). The results were expressed per mg of protein.

### Determination of ADMA chorionic villous tissue

ADMA was determined in tissue lysates from 17 normal and 17 high risk pregnancies using an ELISA as detailed by the manufacturer (DLD Diagnostika, Hamburg, Germany) using the method detailed above for the cGMP assay in chorionic villous tissue.

### Statistical analysis

Student’s t-test and analysis of variance (ANOVA) were performed using GraphPad Prism (version 7.0, CA, USA) or where appropriate Mann-Whitney [18, 19]. Significance was accepted at *p < 0.05.

## Results

### Characterisation of first trimester PEC

**Fig. 1:**
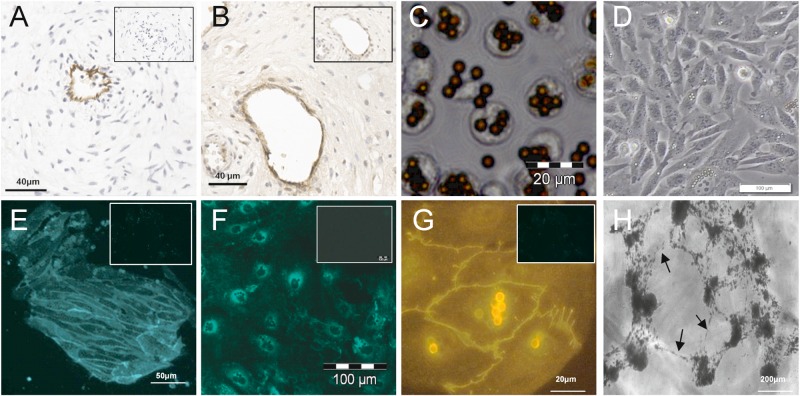
Characterisation of first trimester placental endothelial cells (PEC). Immunohistochemical analysis of **a** CD31 and **b** vWF expression in transverse paraffin-embedded first trimester chorionic villous tissue and are representative of *n* = 3 placental samples. The appropriate immunoglobulin controls are shown in the insert. **c** Freshly isolated first trimester PEC with CD31-coated magnetic beads attached prior to adhesion to the plate. **d** A monolayer culture of PEC 96 h after isolation. **e** A colony of PEC stained for CD31 expression 96 h after isolation. Immunoglobulin isotype control is shown in the insert. **f** vWF expression by PEC 96 h after isolation with the appropriate immunoglobulin control shown in the insert. **g** VE-cadherin expression with control immunoglobulin (insert). **h** PEC formation of endothelial cell network following 6 days growth on collagen. The arrows indicate the  endothelial cell networks characteristic of these cultures

Prior to developing an isolation protocol for first trimester PEC, we examined the expression of endothelial cell markers in placental tissue sections obtained from pregnancies between 9 and 14 weeks gestation. In these sections, positive expression was observed for von Willibrand factor (vWF) and CD31 in cells lining vessels (Fig. 1a, b). We then adapted existing protocols using anti-CD31 coated Dynabeads® for the isolation of first trimester PEC (Fig. 1c). The resulting cells exhibited a classic cobblestone-like appearance (Fig. 1d). CD31 Dynabeads® remained attached to PEC for up to 12 days in culture. The purity of endothelial cell cultures as determined by the expression of CD31 (Fig. 1e) vWF (Fig. 1f) and VE-cadherin (Fig. 1g) after 24 h, and 72 h in culture and was found to be 83.5 ± 1.6% (SEM, *n* = 16) and 82.2 ± 3.2% (SEM, *n* = 7). They were negative for the trophoblast marker cytokeratin 7. The resulting cells expressed and formed an endothelial cell network when grown on collagen (Fig. 1h).

### PEC apoptosis and proliferation

**Fig. 2:**
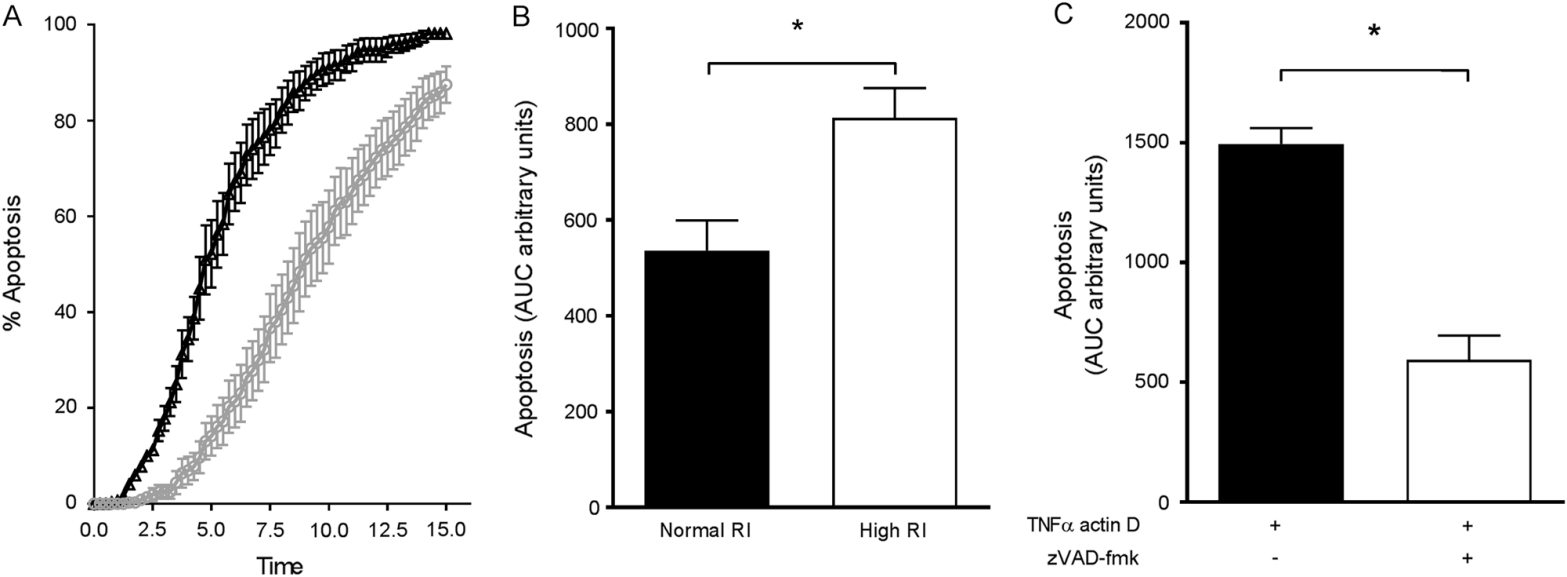
Apoptosis of first trimester PEC from normal- and high-RI pregnancies in response to stimulation with TNFα and actinomycin D. First trimester PEC were isolated from high- and normal-RI patients and cultured with 30 ng/ml TNFα and 800 ng/ml actinomycin D. Images were taken every 15 min over 15 h. **a** The kinetics of the induction of apoptosis for PEC, high-RI (*n* = 8 mean ± SEM, black symbols) and normal-RI (*n* = 8 mean ± SEM, grey symbols). **b** Area under the kinetics curve data from the same patients. **c** In a separate cohort, normal-RI PEC were incubated with 30 ng/ml TNFα and 800 ng/ml actinomycin D (*n* = 4) alone and in the presence of the broad-spectrum caspase inhibitor zVAD-fmk (*n* = 4). The results are expressed as mean ± SEM (*p < 0.05) as determined by Mann-Whitney

The balance between cell death and survival in response to TNFα can be tipped in favour of cell death by inhibiting the expression of various survival factors. To mimic this we examined the sensitivity of PEC isolated from high-RI and normal-RI pregnancies to stimulation with TNFα in the presence of the mRNA synthesis inhibitor, actinomycin D. Apoptosis was assessed by time-lapse microscopy and the kinetics of the induction assessed. After 15 h in culture, 98% of cells from high-RI pregnancies treated with TNFα/actinomycin D had undergone apoptosis (Fig. 2a). The time at which 50% of the cells had died was determined from the kinetics curves. In the high-RI group the t½ was 4.97 ± 0.32 h (*n* = 8) while t½ in the normal-RI group was 9.14 ± 0.86 h (*n* = 8). Expressing the data as the area under the kinetics curve showed that there was a 1.5-fold increase in apoptosis in the high-RI compared to the normal-RI group (Fig. 2b, *p* < 0.01; *n* = 8 in each group). Incubation of PEC with the broad-spectrum caspase inhibitor, zVAD-fmk, prior to the addition of the TNFα/actinomycin D inhibited apoptosis (Fig. 2c, *n* = 4 in each group), indicating that cell death was caspase dependent. Gestational age had no significant effect on the sensitivity to apoptosis found within each group.

**Fig. 3:**
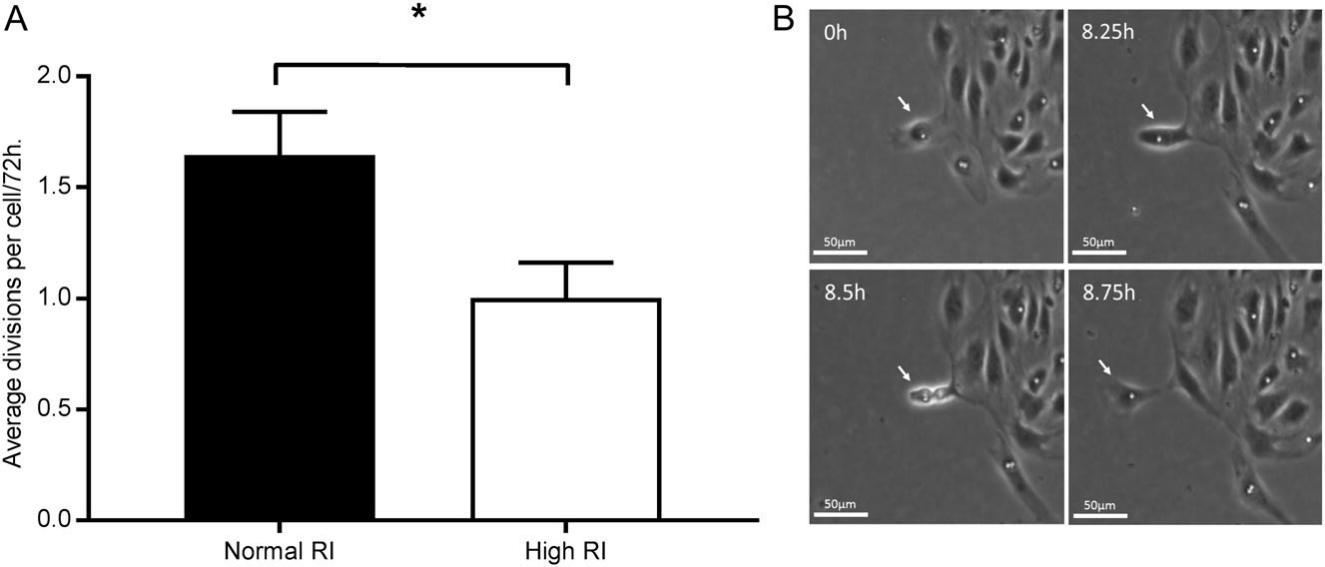
Proliferation of first trimester PEC from normal- and high-RI pregnancies. **a** First trimester PEC were isolated from normal- and high-RI patients. The average number of cell divisions were observed over a 72 h period and the results are expressed as mean ± SEM for *n* = 5 patients per group (*p < 0.05) as determined by the Student’s *t*-test. **b** Four time-lapse images of a normal first trimester PEC undergoing cell division. The dividing cell is indicated by the white arrow

Cell proliferation in the absence of any external stimuli was followed by time-lapse microscopy. Cells associated with at least one CD31-coated bead were followed and the number and time of the cell divisions were then recorded. The results were expressed as the average number of cell divisions per cell. There were significantly more cell divisions in the cells isolated from the normal-RI (*p* < 0.05) compared to the high-RI pregnancies (Fig. 3, *n* = 5 in each group).

**Fig. 4:**
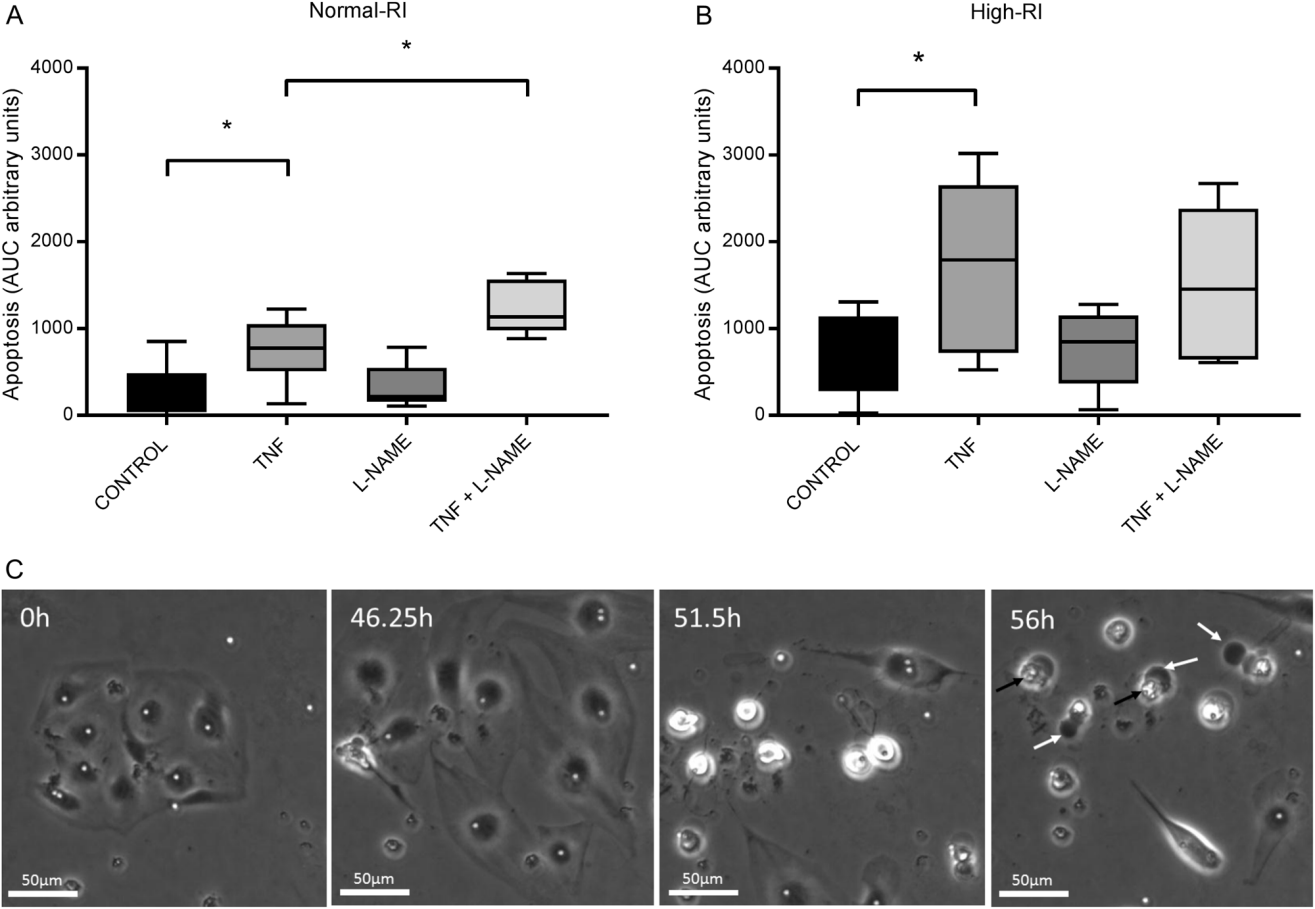
The effect of TNFα alone and in combination with the nitric oxide synthase inhibitor L-NAME on apoptosis of first trimester PEC from normal- and high-RI pregnancies. PEC from normal (**a**) and high-RI (**b**) patients were incubated with either TNFα alone (30 ng/ml), or in combination with 500 µM L-NAME. Images were taken every 15 min over 72 h. Apoptosis was expressed as area under the kinetics curve. The data is presented as a box and whisker plot illustrating the median, the 25th and 75th percentiles and the maximum and minimum values of *n* = 6 normal- and *n* = 5 high-RI patients (*p < 0.05). Statistical analysis data in (**a**, **b**) were analysed using an ANOVA followed by a Sidak’s multiple comparison test. **c** A time-lapse sequence of PEC isolated from a normal pregnancy undergoing apoptosis in response to incubation with TNFα and 500 µM L-NAME. Cells round up and become phase bright (51.5 h) before blebbing (56 h) as indicated by the black arrows and then blistering as indicated by the white arrows

Having identified a difference in the susceptibility of the PEC from high-RI and normal-RI pregnancies to pharmacological stimulation of apoptosis, we repeated these experiments using TNFα alone. TNFα significantly induced apoptosis in PEC isolated from both the normal- and high-RI pregnancies (Fig. 4, *n* = 6 normal- and *n* = 5 high-RI samples) however the effect was significantly greater (>2 fold) in the high-RI compared to the normal-RI group. In the absence of any stimulus there was more basal apoptosis in the PEC from the high-RI compared to the normal-RI pregnancies. In fact the basal rate of apoptosis in the high-RI was equivalent to that seen in the PEC from normal-RI stimulated with TNFα.

NO is an important regulator of endothelial cell survival, we therefore examined whether manipulating the synthesis of NO by PEC could play a role in the differences seen in sensitivity. Inhibition of NO synthesis with l-nitro-arginine-methyl ester (L-NAME) significantly increased the apoptotic response seen in PEC from the normal-RI group treated with TNFα (Fig. 4b, *n* = 6 normal- and *n* = 5 high-RI samples), but there was no further increase in cell death in the high-RI cells (Fig. 4c).

**Fig. 5:**
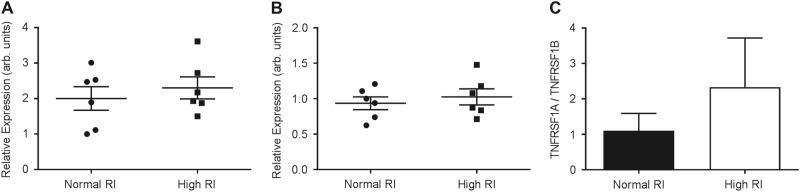
TNFα receptor gene expression in first trimester PEC isolated from normal- and high-RI pregnancies. Quantitative Real Time PCR (qRT-PCR**)** was performed on first trimester PEC that were isolated from normal- and high-RI patients after 96 h in culture for **a** TNFRSF1A and **b** TNFRSF1B. **c** the ratio of TNFRSF1A/TNFRSF1B. The results are presented as relative expression in arbitrary units and are the means ± SEM of *n* = 6 normal and *n* = 6 high-RI patients

We have previously shown that NO can regulate the expression of apoptotic receptors [20]. To investigate this possibility further we examined the expression of two isoforms of the TNF-receptor family, TNFRSF1A and TNFRSF1B. We found no difference in the expression of mRNA for either receptor isoform between the two groups (Fig. 5a, b, *n* = 6 in each group). There was also no difference in the ratio of expression between the two isoforms in the two groups (Fig. 5c).

### Nitric oxide expression and activity

**Fig. 6:**
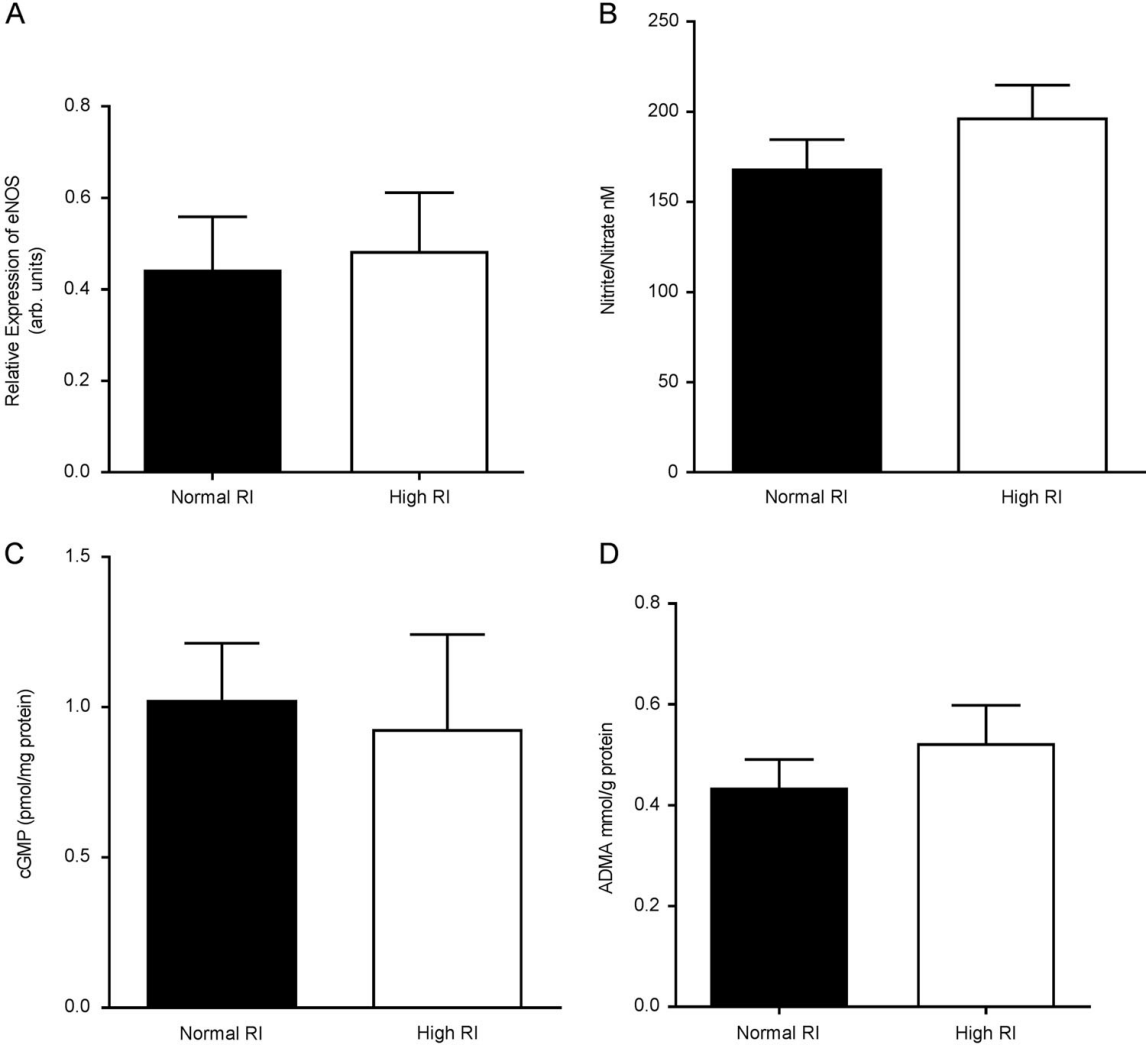
Nitric oxide expression and activity in first trimester PEC and chorionic villous tissue. **a** PEC were isolated and the mRNA extracted immediately. NOS3 gene expression was determined by qRT-PCR (*n* = 7 for each normal- and high-RI). **b** Nitrite/nitrate (*n* = 30 normal- and *n* = 37 high-RI) and **c** NO production was indirectly determined by assessment of the production of cGMP (*n* = 23 normal- and *n* = 16 high-RI) **d** The concentration of the endogenous inhibitor of nitric oxide synthesis, ADMA, in first trimester PEC determined by ELISA (*n* = 17 normal- and *n* = 17 high-RI)

We determined the expression of NOS3 by PCR in PEC cultured for 96 h. There was no difference in the expression of NOS3 mRNA (Fig. 6a, *n* = 7 in each group). Paucity of cells precluded the measurement of NOS activity or changes in phosphorylation status of NOS3 in isolated cells, we therefore measured the production of nitrite/nitrate (NOx) (Fig. 6b, *n* = 30 normal- and *n* = 37 high-RI samples) and cGMP (Fig. 6c, *n* = 23 normal- and *n* = 16 high-RI samples) as surrogate markers of NOS activity in chorionic tissue extracts from high- and normal-RI pregnancies. There was no difference in the content of either cGMP or NOx. We also measured the concentration of asymmetric dimethyl-arginine (ADMA), an endogenous inhibitor of NOS, but found no difference (Fig. 6d, *n* = 17 in each group).

## Discussion

Stereological assessment at term provides some insight into placental angiogenic processes while 3D vascular casts and reconstructions have identified differences in the vasculature between normal pregnancies and those with placental insufficiency [4]. In pregnancies complicated by FGR there is a reduction in vascular branching and elongation of the terminal villous vasculature together with a reduction in capillary volume, surface area and length [21, 22]. However it is not clear whether the differences seen at term are due to inherent differences in the PEC from these pregnancies or are merely a consequence of the placental insufficiency. Using UtAD as a proxy measure of poor placentation in the first trimester we have examined aspects of PEC biology that may contribute to the poor vascular development and hypothesised that PEC derived from high-RI pregnancies would be more sensitive to apoptotic stimuli than those isolated from pregnancies with a normal-RI.

The balance between cell survival and death plays a significant role in aspects of normal development including placental angiogenesis. Factors that upset or alter this balance may therefore be responsible for the poor vascular development seen in pregnancies complicated by early onset PE and FGR [23]. The data presented here indicates that PEC from high-RI pregnancies grow more slowly than those isolated from normal-RI pregnancies. Although this may reflect a differential growth factor receptor expression between the two groups we have not yet identified the pathways responsible, and this will be the focus of further studies.

It has previously been shown that PEC isolated at term from pre-eclamptic pregnancies were less viable than those from normotensive pregnancies [24], while others have reported the increased expression of apoptosis related genes in term placental tissue from pregnancies complicated by PE and FGR [23]. In our initial studies, cells from both the normal- and high-RI pregnancies were sensitive to stimulation by TNFα in the presence of the mRNA synthesis inhibitor actinomycin D, with cells isolated from high-RI pregnancies being more susceptible to induced cell death than those from normal-RI pregnancies. Using the non-specific caspase inhibitor, z-vad.fmk, the mechanism of cell death was identified as apoptosis. To address whether PEC from high-RI pregnancies were more sensitive to apoptotic stimuli under more physiological circumstances the experiments were repeated in the presence and absence of TNFα alone. PEC from high-RI pregnancies were significantly more sensitive to TNFα than those isolated from normal-RI pregnancies. In the absence of TNFα there was over twice as much apoptotic cell death in the high-RI compared to normal-RI pregnancies, although this did not reach statistical significance. There was also no difference in the extent of apoptosis seen in normal-RI cells treated with TNFα compared to untreated PEC from high-RI patients (Fig. 4a, b). Collectively this suggests that cells from the high-RI group were inherently more susceptible to apoptosis and more sensitive to apoptotic stimulation than those from the normal-RI pregnancies.

TNFα can exert two opposing biological effects; a pro-inflammatory, pro-survival effect through the activation of NF-κB and pro-apoptotic effects through the activation of caspase-8. TNFα binds to two receptors, TNFR1 and TNFR2 encoded by the two genes TNFRSF1A and TNFRSF1B respectively and both are expressed by PEC. TNFR2 mediates pro-survival pathways while TNFR1 is involved in both the pro-inflammatory and pro-apoptotic pathways. The pathway taken is dependent on the cellular and environmental context. We therefore examined the expression of TNFRSF1A and TNFRSF1B gene expression, however found no significant difference in receptor expression between the two groups. Although these results exclude differential receptor expression as the mechanism underlying the difference in sensitivity, it does not exclude differences in post-receptor mechanisms such as caspase activation.

NO is an important inter and intra cellular signalling molecule that plays a pivotal role in EC survival and function. We and others have shown that NO can inhibit apoptosis in a number of cell types through post-translational modification of key apoptotic proteins. One such mechanism, nitrosylation, is a reversible modification of cysteine residues that has been shown to directly inhibit caspase activity by modification of the key cysteine residue in the active site [13, 25, 26]. NO has also been implicated in the indirect inhibition of apoptosis by preventing the phosphorylation of cFLIP by protein kinase ε^13^. In addition to the direct effect of NO on protein activity the actions of NO can also be mediated through the activation of soluble guanylate cyclase, the generation of cGMP and the activation of protein kinase G.

NO is synthesised by a family of enzymes the NO-synthases (NOS). The most prominent NOS expressed by endothelial cells is NOS3. NOS3 is activated by calcium ions and regulated by phosphorylation. We assessed the involvement of NO in the regulation of PEC apoptosis a`t both a functional level using inhibitors of NO synthesis and at a biochemical level examining NOS3 activity indirectly via changes in cGMP, nitrite/nitrate and the production of endogenous inhibitors. We found that there was no difference in the production of either cGMP or nitrate/nitrite by chorionic villous tissue, two downstream markers of NOS activity. NOS activity can also be regulated by the endogenous inhibitor of NO synthesis, asymmetric dimethylarginine (ADMA). We have previously shown that the circulating concentration of this inhibitor is elevated in pregnancies complicated with PE [27]. However, again no differences were found between the two groups. Importantly though, we were able to demonstrate a functional role for NO in this system as inhibition of NO synthesis by L-NAME significantly increased the sensitivity of PEC to TNFα induced apoptosis in cells isolated from the normal-RI but not the high-RI group.

In conclusion, we have for the first time demonstrated biological differences in PEC isolated from pregnancies with normal and poor placentation in the first trimester. Specifically we have shown that PEC isolated from pregnancies at increased risk of developing early onset FGR and PE have a reduced proliferation rate and are more prone to apoptosis. Further, we have shown that these cells are more sensitive to the induction of apoptosis in response to TNFα and this may involve the production of NO. Their sensitivity to apoptotic stimuli may contribute to the poor vascular development seen in these pregnancies at term. A better understanding of the underlying mechanisms contributing to this may lead in the future to interventions to improve placental vascular development and therefore pregnancy outcomes.
